# Characterization of Cellulolytic Bacterial Cultures Grown in Different Substrates

**DOI:** 10.1155/2013/689235

**Published:** 2013-11-11

**Authors:** Mohamed Idris Alshelmani, Teck Chwen Loh, Hooi Ling Foo, Wei Hong Lau, Awis Qurni Sazili

**Affiliations:** ^1^Department of Animal Science, Faculty of Agriculture, Universiti Putra Malaysia (UPM), 43400 Serdang, Malaysia; ^2^Department of Animal Production, Faculty of Agriculture, University of Sabha, Sabha, Libya; ^3^Institute of Tropical Agriculture, Universiti Putra Malaysia (UPM), 43400 Serdang, Malaysia; ^4^Department of Bioprocess Technology, Faculty of Biotechnology and Biomolecular Sciences, UPM, 43400 Serdang, Malaysia; ^5^Institute of Bioscience, Universiti Putra Malaysia (UPM), 43400 Serdang, Malaysia; ^6^Department of Plant Protection, Faculty of Agriculture, Universiti Putra Malaysia (UPM), 43400 Serdang, Malaysia

## Abstract

Nine aerobic cellulolytic bacterial cultures were obtained from the Leibniz Institute DSMZ-German Collection of Microorganisms and Cell Culture (DSMZ) and the American Type Culture Collection (ATCC). The objectives of this study were to characterize the cellulolytic bacteria and to determine the optimum moisture ratio required for solid state fermentation (SSF) of palm kernel cake (PKC). The bacteria cultures were grown on reconstituted nutrient broth, incubated at 30°C and agitated at 200 rpm. Carboxymethyl cellulase, xylanase, and mannanase activities were determined using different substrates and after SSF of PKC. The SSF was conducted for 4 and 7 days with inoculum size of 10% (v/w) on different PKC concentration-to-moisture ratios: 1 : 0.2, 1 : 0.3, 1 : 0.4, and 1 : 0.5. Results showed that *Bacillus amyloliquefaciens* 1067 DSMZ, *Bacillus megaterium* 9885 ATCC, *Paenibacillus curdlanolyticus* 10248 DSMZ, and *Paenibacillus polymyxa* 842 ATCC produced higher enzyme activities as compared to other bacterial cultures grown on different substrates. The cultures mentioned above also produced higher enzyme activities when they were incubated under SSF using PKC as a substrate in different PKC-to-moisture ratios after 4 days of incubation, indicating that these cellulolytic bacteria can be used to degrade and improve the nutrient quality of PKC.

## 1. Introduction

Agrowaste features high concentrations of nonstarch polysaccharides (NSPs) such as mannan, xylan, and cellulose. These molecules cannot be digested by monogastric animals and need to be decomposed by cellulolytic microorganisms. The most common agrowaste produced in Malaysia is palm kernel cake (PKC). This by-product is produced as a result of oil extraction from palm fruits. The concentration of NSPs in PKC is 78% mannan, 3% arabinoxylan, 3% glucuronoxylan, and 12% cellulose [1]. Therefore, *β*-mannanase, cellulase, xylanase [2, 3], *α*-galactosidase [1], and *β*-mannosidase [4] can be applied to degrade PKC effectively.

Recently, cellulolytic microorganisms have been used to produce specific enzymes through fermentation technology using agrobyproducts as substrates [2, 3]. Additionally, the nutritive quality of agrobyproduct can be enhanced through solid state fermentation (SSF) and used as animal feed stuff [5, 6]. SSF is a technology that can be defined as the growth of a microorganism in solid substrate containing moisture in a ratio ranging from 1 : 0.1 to 1 : 1 [7]. The advantages of using a low moisture level in SSF include reduced opportunities for contamination by other microorganisms and improved aeration as well as increased porosity between particles [8]. In contrast, the high level of moisture in the substrate during SSF leads to increased chances for contamination by other microbes and reduced aeration as well as decreased porosity between particles. Moreover, it causes agglomeration and a gummy substrate texture. Thus, the problems associated with heat and oxygen transfer would occur [9–11]. 

Usually, the substrate-to-moisture ratio in SSF mediated by cellulolytic bacteria culture ranges between 1 : 0.1 and 1 : 1 [12]. However, some bacterial cultures require a higher level of moisture for enzyme production or substrate degradation. For instances, increased xylanase production by *B. licheniformis *A99 [9] and *B. pumilus *[10] occurred during SSF with a moisture ratio of 1 : 2.5 (w/v), whereas a high level of xylanase production was observed for *Bacillus *sp. AR-009 during SSF with a moisture ratio of 1 : 0.5 to 1 : 1.5 (w/v) [11]. In comparison, the optimum substrate-to-moisture ratio was reported to be 1 : 0.75 (w/v) for fungus culture of *Aspergillus niger *USM AI1 grown in PKC [13] and 1 : 0.85 (w/v) for *B. amyloliquefaciens *grown in groundnut oil cake mixed with wheat bran [10].

As for the effect of inoculum size, 10% (v/w) was reported to be the optimal inoculum size of SSF for alkaliphilic *Bacillus *sp. [11], *B. megaterium *[8], and *B. pumilus *[10]. However, the optimum inoculum size was reported to be 10 to 15% (v/w) for xylanase production by thermophilic *B. licheniformis *A99 during SSF [9]. Therefore, reducing the moisture content during SSF process is essential [14]. In addition, the inoculum size should be sufficient to decrease the possibility of contamination during SSF process.

PKC demonstrates high levels of NSPs; the nutritive quality of this agro-byproduct can be improved by cellulolytic microbes through SSF. Although fungi have many characteristics and produce higher enzyme activity than bacteria, the secondary products of fungi, such as mycotoxins would depress the growth of animals. The mycotoxin problem can be overcome by replacing fungi with cellulolytic bacteria in SSF. Furthermore, information concerning the enhancement of nutritive values of PKC through SSF using *Bacillus glucanolyticus *DSMZ 5162, *B. amyloliquefaciens *DSMZ 1067, *Cellulomonas fimi *DSMZ 20114, *Paenibacillus curdlanolyticus *DSMZ 10248, *P. polymyxa *ATCC 842, *B. circulans *ATCC 61, *B. megaterium *ATCC 9885, *B. wakoensis *DSMZ 2512, and *B. cellulosilyticus *DSMZ 2522 has been reported elsewhere. Thus, the objectives of this study were to characterize different cellulolytic bacteria with different substrates, such as carboxymethyl cellulose (CMC), xylan, and locust bean gum (LBG) galactomannan and to determine the optimum PKC : moisture ratio for the SSF mediated by cellulolytic bacteria.

## 2. Materials and Methods

### 2.1. Organisms and Growth Conditions

Nine aerobic cellulolytic bacterial cultures were purchased from DSMZ and ATCC. They were *Bacillus glucanolyticus *DSMZ 5162, *Bacillus amyloliquefaciens *DSMZ 1067, *Cellulomonas fimi *DSMZ 20114, *Paenibacillus curdlanolyticus *DSMZ 10248, *Paenibacillus polymyxa *ATCC 842, *Bacillus circulans *ATCC 61, *Bacillus megaterium *ATCC 9885,* Bacillus wakoensis *DSMZ 2512, and *Bacillus cellulosilyticus *DSMZ 2522. 

The bacteria cultures were grown in nutrient broth and agar containing (g/L) peptone, 15.0; sodium chloride, 6.0; yeast extract, 3.0; agar-agar, 12.0; and glucose, 1.0 at pH 7.0. However, *B. wakoensis *DSMZ 2512 and *B. cellulosilyticus *DSMZ 2522 were grown in alkaline nutrient broth at pH 9. The glucose of nutrient broth and agar was substituted with CMC, xylan from Birchwood, or LBG galactomannan as a carbon source to determine the activity of CMCase (cellulase), xylanase, and mannanase, respectively. The bacteria cultures were incubated at 30°C and agitated at 200 rpm in a rotary shaker to prepare the working inoculum. 

### 2.2. Cellulolytic Enzyme Production in SSF

After being ground, sieved, and dried overnight at 60°C, 5 g of PKC was transferred to 150 mL conical flasks. Distilled water was added to the PKC to obtain PKC : moisture ratios of 1 : 0.2, 1 : 0.3, 1 : 0.4, and 1 : 0.5. The flasks were then stoppered with a cotton plug and autoclaved at 121°C for 30 min. The flasks were then cooled to room temperature and inoculated with 10% (v/w) inocula (0.5 mL/5 g). Finally, the conical flasks were incubated at 30°C for 4 and 7 days under humidified conditions created by placing sterile distilled water inside the incubator. The best bacterial cultures that produced higher enzyme activity were cultured again with PKC : moisture ratios of 1 : 0.2, 1 : 0.4, 1 : 0.6, 1 : 0.8, and 1 : 1 in order to determine the optimum moisture ratio for each bacteria culture.

### 2.3. Extraction of Crude Enzyme

The bacteria cultures were revived in media containing CMC, xylan, or LBG galactomannan for 9 days in order to obtain the working inoculum. The crude enzyme was extracted by centrifugation at 10,000 g, 4°C for 15 min. The clear supernatant was considered to be the crude enzyme, and enzyme activity was determined for each culture grown in different substrates.

Extraction of crude enzyme during SSF was accomplished by adding 20 mL sterile distilled water into each conical flask and agitated overnight at 25°C on a rotary shaker at 130 rpm. The solution was filtrated using Whatman paper no. 1 and then centrifuged at 10,000 g, 4°C for 15 min. The supernatant was kept at −20°C for further analysis.

### 2.4. Enzyme Activity Assay

Mannanase, xylanase, and CMCase activities were determined according to the modified methods of Araujo and Ward [15], Bailey et al. [16], and Miller [17], respectively. Standard references were plotted for mannose, xylose, and glucose, and the absorbance was read using a spectrophotometer at 540 nm.

The soluble protein was determined [18] to calculate the specific enzyme activity for each bacteria culture, and bovine serum albumin (BSA) was used as a standard. All enzyme activities were assayed in triplicate, and the average enzyme activity was presented as *μ*mol/min/mg protein. The enzyme activity is defined as the ability of enzyme to release one *μ*mol of reducing sugar per minute in specific conditions.

### 2.5. Statistical Analysis

Data were analyzed using two-way ANOVA for the moisture ratio treatments, and the treatment means, which showed significant differences at a probability level of 0.05, were separated by Tukey's test using general linear model (GLM) procedure of Statistical Analysis System [19].

## 3. Results and Discussion

**Figure 1:**
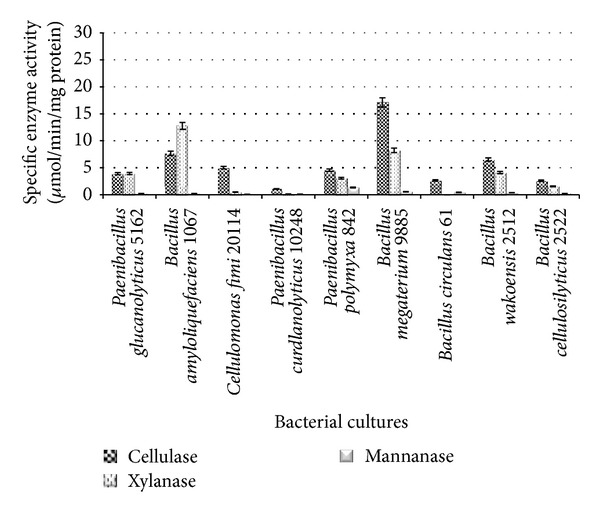
Specific CMCase, xylanase, and mannanase activities of cellulolytic bacteria grown in CMC medium.

The characteristics of cellulolytic bacterial cultures in CMC medium are shown in Figure 1. The highest cellulase activity was observed in *B. megaterium* and *B. amyloliquefaciens* (17.11 and 7.69 *μ*mol/min/mg protein, resp.), whereas the highest xylanase activity was 12.76 and 8.23 *μ*mol/min/mg protein, produced by *B. amyloliquefaciens* and *B. megaterium*, respectively (Figure 1).

**Figure 2:**
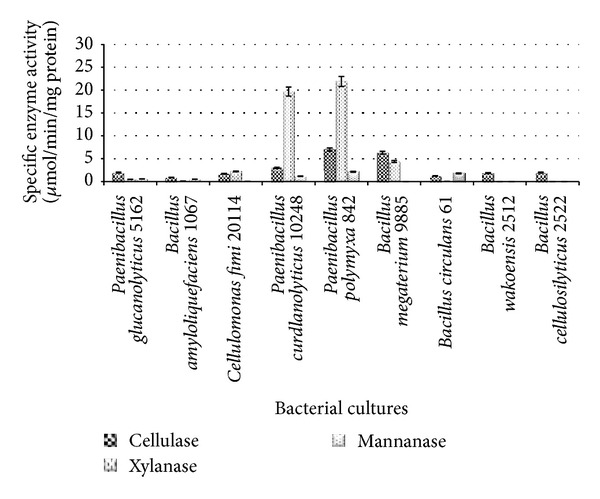
Specific CMCase, xylanase, and mannanase activities of cellulolytic bacteria grown in xylan medium.

The characteristics of cellulolytic bacterial cultures in xylan medium are shown in Figure 2. *P. polymyxa *ATCC 842 and *P. curdlanolyticus *DSMZ 10248 produced higher xylanase activity (21.9 and 19.67 *μ*mol/min/mg protein, resp.) compared to the other bacterial cultures (Figure 2).

The findings are in agreement with [20, 21], who showed that *P. curdlanolyticus* produced multienzyme complexes and that xylanase activity was the main enzyme produced.

**Figure 3:**
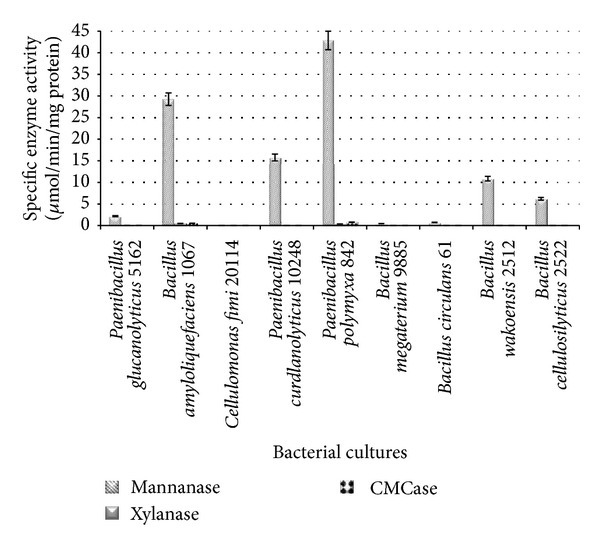
Specific CMCase, xylanase, and mannanase activities of cellulolytic bacteria grown in LBG medium.

The cellulolytic bacterial cultures characterized in LBG medium are shown in Figure 3. The highest mannanase activity was observed with *P. polymyxa *ATCC 842, *B. amyloliquefaciens* DSMZ 1067, and *P. curdlanolyticus *DSMZ 10248 (42.85, 29.27, and 15.77 *μ*mol/min/mg protein, resp.) as compared to the other bacterial cultures (Figure 3).

The high mannanase production could be a result of the ability of these bacterial cultures to degrade mannan. These findings are in agreement with [22], who reported that *B. amyloliquefaciens *was capable of degrading galactomannan. In addition, the findings are in agreement with other studies [23, 24] indicating that *P. curdlanolyticus* was capable of producing a multienzyme complex.

It appears that *B. amyloliquefaciens* DSMZ 1067 and *P. polymyxa *ATCC 842 can be considered multifunctional enzyme producers because of their abilities to produce higher enzyme activity in both CMC and LBG mediums. This finding is in agreement with those of Mabrouk and El Ahwany [22], who screened *B. amyloliquefaciens *in galactomannan medium. In addition, *P. polymyxa *ATCC 842 is able to produce multienzymes such as *β*-1,3 and *β*-1,6 glucanase [25], cellulase, xylanase, lichenase, and mannanase [22]. *P. curdlanolyticus *DSMZ 10248 appears to be xylanolytic bacteria because of its capability to produce xylanase and mannanase in xylan and mannan mediums. Sudo et al. [26] reported that the genus *Paenibacillus *was capable of degrading various polysaccharides and able to secrete multifunctional enzymes, mainly xylanases. The results of this study are also consistent with previous findings concerning *P. curdlanolyticus*, which has been shown to exhibit effective degradation of xylan and cellulose and to produce a multienzyme complex containing several xylanases and cellulases [24].

The production of cellulolytic enzymes under SSF for 4 and 7 days with different PKC : moisture ratios is shown in Table 1.

The highest cellulolytic enzyme activities were significantly (*P* < 0.05) exhibited by *B. megaterium *ATCC 9885, *P. curdlanolyticus *DSMZ 10248, and *P. polymyxa *ATCC 842 as compared to the other bacterial cultures. The production of enzymes significantly (*P* < 0.05) declined at the 7th day of SSF, and the highest production was observed in *B. megaterium *ATCC 9885, *P. curdlanolyticus *DSMZ 10248, and *P. polymyxa *ATCC 842. The dramatic decline of cellulolytic enzyme production could be due to the depletion of the carbon source after 7 days of SSF.

**Figure 4:**
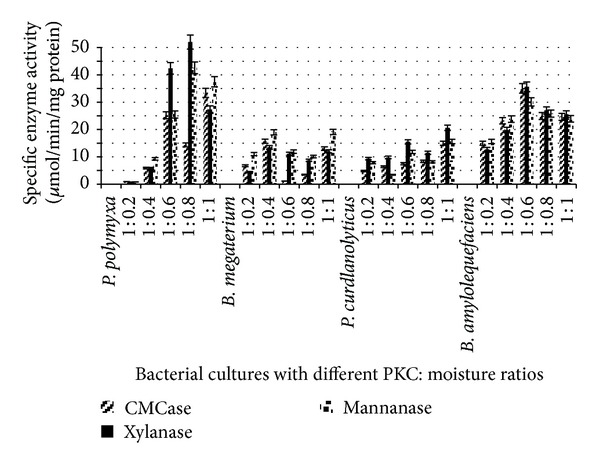
Specific enzyme activity for some bacterial cultures under SSF with different PKC : moisture ratios.

The optimum PKC : moisture ratio appeared to be 1 : 0.8, 1 : 0.4, 1 : 1, and 1 : 0.6 (w/v) during SSF for *P. polymyxa *ATCC 842, *B. megaterium *ATCC 9885, *P. curdlanolyticus *DSMZ 10248, and *B. amyloliquefaciens *DSMZ 1067, respectively (Figure 4). 

The enzyme activity was higher than the other ratios during SSF for 4 days. These findings are consistent with the findings of Gessesse and Mamo [11], who claimed that *Bacillus *sp. produced high enzyme activity when the moisture ratios ranged from 1 : 0.5 to 1 : 1.5 (w/v). The moisture content in the substrate can be considered as an important factor in SSF and microbial growth. A ratio greater than the optimum level may decrease the porosity of the substrate, produce a substrate with a gummy texture, and lower the oxygen transfer rate. However, a lower moisture level than the optimum ratio could limit the growth of the microorganism in the substrate [8–11, 27]. 

## 4. Conclusions

The bacterial cultures that exhibited the ability to degrade different substrates were *B. megaterium *ATCC 9885, *P. curdlanolyticus *DSMZ 10248, *P. polymyxa *ATCC 842, and *B. amyloliquefaciens *DSMZ 1067. In addition, the best moisture ratios were 1 : 0.8, 1 : 0.4, 1 : 1, and 1 : 0.6 (w/v) for *P. polymyxa *ATCC 842, *B. megaterium *ATCC 9885, *P. curdlanolyticus *DSMZ 10248, and *B. amyloliquefaciens *DSMZ 1067, respectively, after 4 days of incubation during SSF. Based on the results obtained, these cellulolytic bacteria can be used to degrade and improve the nutrient quality of the PKC by eliminating crude fibers.

## Figures and Tables

**Table 1:** Production of cellulolytic enzymes from bacterial cultures under SSF for 4 and 7 days with different PKC : moisture ratios.

Bacterial culture	Moisture ratio	CMCase		Xylanase		Mannanase	
		4 days	7 days	4 days	7 days	4 days	7 days
*B. amyloliquefaciens *DSMZ	1 : 0.2	3.91 ± 0.060^fgh^	1.66 ± 0.045^ghi^	3.32 ± 0.023^f^	1.39 ± 0.082^fg^	4.07 ± 0.030^f^	2.08 ± 0.184^fgh^
	1 : 0.3	3.72 ± 0.007^gh^	1.12 ± 0.084^ij^	3.20 ± 0.143^f^	1.10 ± 0.045^ghi^	3.83 ± 0.054^f^	1.49 ± 0.126^hij^
	1 : 0.4	3.29 ± 0.033^ghi^	1.26 ± 0.096^hij^	2.81 ± 0.032^f^	1.15 ± 0.130^ghi^	3.37 ± 0.042^fgh^	1.48 ± 0.070^hij^
	1 : 0.5	3.50 ± 0.012^gh^	0.20 ± 0.047^mno^	3.08 ± 0.018^f^	0.49 ± 0.127^jklmn^	3.58 ± 0.022^fg^	0.89 ± 0.006^jkl^

*P. curdlanolyticus *DSMZ	1 : 0.2	4.84 ± 2.300^efg^	0.26 ± 0.150^mno^	9.36 ± 0.360^c^	0.25 ± 0.130^klmno^	7.87 ± 2.100^d^	0.78 ± 0.340^klm^
	1 : 0.3	5.40 ± 0.440^def^	0.10 ± 0.030^no^	9.74 ± 0.070^c^	0.13 ± 0.030^mno^	4.82 ± 1.270^ef^	0.78 ± 0.280^klm^
	1 : 0.4	6.38 ± 0.310^d^	2.16 ± 0.290^efg^	9.76 ± 0.370^c^	1.13 ± 0.280^ghi^	3.43 ± 1.420^fgh^	0.28 ± 0.140^mno^
	1 : 0.5	10.40 ± 1.00^d^	5.70 ± 0.030^c^	14.35 ± 0.300^b^	6.67 ± 0.750^b^	8.82 ± 1.250^cd^	5.35 ± 1.140^d^

*P. polymyxa *ATCC	1 : 0.2	0.93 ± 0.112^lmno^	2.50 ± 0.204^e^	0.73 ± 0.006^ij^	2.36 ± 0.161^de^	0.80 ± 0.024^jkl^	2.15 ± 0.112^fgh^
	1 : 0.3	0.62 ± 0.014^mnop^	0.91 ± 0.014^jkl^	0.48 ± 0.015^jk^	0.78 ± 0.002^ghij^	0.71 ± 0.031^jkl^	0.92 ± 0.074^jkl^
	1 : 0.4	2.12 ± 0.303^ijk^	2.53 ± 0.131^e^	2.10 ± 0.044^g^	2.10 ± 0.027^e^	3.33 ± 0.580^fgh^	2.77 ± 0.547^f^
	1 : 0.5	6.45 ± 0.035^d^	3.85 ± 0.110^d^	6.30 ± 0.016^d^	3.08 ± 0.300^cd^	10.01 ± 0.363^cd^	3.68 ± 0.083^e^

*B. megaterium *ATCC	1 : 0.2	6.74 ± 0.570^d^	3.46 ± 0.070^d^	4.42 ± 0.080^e^	3.53 ± 0.940^c^	11.03 ± 3.760^c^	4.10 ± 0.590^e^
	1 : 0.3	5.82 ± 0.270^de^	8.77 ± 0.220^b^	4.98 ± 0.005^e^	7.43 ± 0.030^b^	7.11 ± 0.010^de^	9.40 ± 0.090^c^
	1 : 0.4	15.68 ± 0.230^b^	12.76 ± 0.170^a^	13.48 ± 0.020^b^	10.80 ± 1.41^a^	18.96 ± 0.440^b^	13.91 ± 0.050^a^
	1 : 0.5	31.24 ± 0.430^a^	11.60 ± 0.110^a^	25.34 ± 1.01^a^	9.77 ± 0.260^a^	35.99 ± 0.020^a^	12.15 ± 0.090^b^

*P. glucanolyticus *DSMZ	1 : 0.2	0.00 ± 0.000^p^	0.00 ± 0.000^o^	0.00 ± 0.000^m^	0.00 ± 0.000^o^	0.00 ± 0.000^l^	0.00 ± 0.000^0^
	1 : 0.3	0.00 ± 0.000^p^	0.00 ± 0.000^o^	0.00 ± 0.000^m^	0.00 ± 0.000^o^	0.00 ± 0.000^l^	0.00 ± 0.000^o^
	1 : 0.4	1.80 ± 0.220^jkl^	0.28 ± 0.004^mno^	1.38 ± 0.100^h^	0.16 ± 0.008^mno^	1.75 ± 0.080^hijk^	0.33 ± 0.030^mno^
	1 : 0.5	1.20 ± 0.060^klmno^	1.00 ± 0.002^jkl^	0.79 ± 0.050^ij^	0.73 ± 0.002^hijk^	1.17 ± 0.030^jk^	1.12 ± 0.080^jk^

*C. fimi *DSMZ	1 : 0.2	1.16 ± 0.160^klmno^	0.51 ± 0.010^lmn^	0.06 ± 0.030^lm^	0.60 ± 0.250^ijklm^	1.05 ± 0.040^jkl^	1.94 ± 0.120^gh^
	1 : 0.3	0.87 ± 0.260^lmno^	0.98 ± 0.680^jkl^	0.16 ± 0.040^lm^	1.29 ± 0.130^fgh^	1.80 ± 0.060^ghijk^	0.67 ± 0.390^klmn^
	1 : 0.4	1.35 ± 0.390^klmn^	1.29 ± 0.030^hij^	0.78 ± 0.290^ij^	1.96 ± 0.060^ef^	3.50 ± 0.360^fgh^	2.48 ± 0.040^fg^
	1 : 0.5	3.52 ± 0.030^gh^	1.79 ± 0.280^fgh^	1.80 ± 0.080^gh^	2.10 ± 0.160^e^	3.36 ± 0.100^fgh^	1.83 ± 0.020^ghi^

*B.circulans *ATCC	1 : 0.2	0.00 ± 0.000^p^	0.00 ± 0.000^o^	0.00 ± 0.000^m^	0.00 ± 0.000^o^	0.75 ± 0.500^jkl^	0.00 ± 0.000^o^
	1 : 0.3	0.47 ± 0.200^nop^	0.38 ± 0.130^mno^	0.70 ± 0.400^ij^	0.00 ± 0.000^o^	1.41 ± 0.090^ijk^	0.05 ± 0.008^o^
	1 : 0.4	0.55 ± 0.310^mnop^	0.53 ± 0.070^klmn^	0.94 ± 0.020^i^	0.26 ± 0.120^jklmno^	1.94 ± 0.130^ghij^	0.20 ± 0.150^no^
	1 : 0.5	2.78 ± 0.040^hij^	0.60 ± 0.320^klm^	1.47 ± 0.030^h^	0.69 ± 0.020^hijkl^	3.60 ± 0.610^fg^	0.78 ± 0.080^klmn^

*B. wakoensis *DSMZ	1 : 0.2	1.42 ± 0.080^klm^	0.41 ± 0.270^mno^	0.29 ± 0.060^kl^	0.10 ± 0.020^mno^	0.69 ± 0.030^jkl^	0.29 ± 0.050^mno^
	1 : 0.3	2.05 ± 0.020^ijk^	2.40 ± 0.180^ef^	0.56 ± 0.020^jk^	0.26 ± 0.030^jklmno^	1.28 ± 0.050^jk^	1.18 ± 0.020^ijk^
	1 : 0.4	1.63 ± 0.030^jkl^	1.02 ± 0.020^jk^	0.52 ± 0.040^jk^	0.22 ± 0.003^klmno^	0.97 ± 0.040^jkl^	0.27 ± 0.040^mno^
	1 : 0.5	2.17 ± 0.009^ijk^	1.65 ± 0.020^ghi^	0.77 ± 0.020^ij^	0.03 ± 0.004^no^	1.38 ± 0.020^ijk^	0.99 ± 0.040^jk^

*B. cellulosilyticus *DSMZ	1 : 0.2	0.00 ± 0.000^p^	0.00 ± 0.000^o^	0.00 ± 0.000^m^	0.00 ± 0.000^o^	0.00 ± 0.000^l^	0.00 ± 0.000^o^
	1 : 0.3	0.47 ± 0.002^nop^	0.00 ± 0.000^o^	0.32 ± 0.020^kl^	0.00 ± 0.000^o^	0.49 ± 0.030^kl^	0.00 ± 0.000^o^
	1 : 0.4	0.38 ± 0.030^op^	0.00 ± 0.000^o^	0.47 ± 0.050^jk^	0.00 ± 0.000^o^	0.73 ± 0.010^jkl^	0.00 ± 0.000^o^
	1 : 0.5	1.86 ± 0.160^jkl^	0.38 ± 0.020^mno^	0.78 ± 0.080^ij^	0.21 ± 0.008^lkmno^	3.00 ± 0.050^fghi^	0.42 ± 0.002^lmno^

^a–p^Means ± SE. Means with different superscripts within the same column are significantly different (*P* < 0.05).
